# Cytoplasmic Incompatibility Variations in Relation with *Wolbachia cid* Genes Divergence in Culex pipiens

**DOI:** 10.1128/mBio.02797-20

**Published:** 2021-02-09

**Authors:** Mathieu Sicard, Alice Namias, Marco Perriat-Sanguinet, Eric Carron, Sandra Unal, Mine Altinli, Frederic Landmann, Mylène Weill

**Affiliations:** a ISEM, University of Montpellier, CNRS, IRD, Montpellier, France; b CRBM, University of Montpellier, CNRS, Montpellier, France; EPFL

**Keywords:** *Culex pipiens*, toxin-antitoxin system, *Wolbachia*, developmental biology, endosymbionts, gene amplification, vectors

## Abstract

In arthropods, *Wolbachia* endosymbionts induce conditional sterility, called cytoplasmic incompatibility (CI), resulting from embryonic lethality. CI penetrance (i.e., embryonic death rate) varies depending on host species and *Wolbachia* strains involved. All Culex pipiens mosquitoes are infected by the endosymbiotic alphaproteobacteria *Wolbachia w*Pip. CI in *Culex*, characterized as a binary “compatible/incompatible” phenomenon, revealed an unparalleled diversity of patterns linked to the amplification-diversification of *cidA* and *cidB* genes. Here, we accurately studied CI penetrance variations in the light of *cid* genes divergence by generating a C. pipiens compatibility matrix between 11 lines hosting different phylogenetic *w*Pip groups and exhibiting distinct *cid* gene repertoires. We showed, as expected, that crosses involving *w*Pip from the same group were mostly compatible. In contrast, only 22% of the crosses involving different *w*Pip groups were compatible, while 54% were fully incompatible. For the remaining 24% of the crosses, “intermediate” compatibilities were reported, and a cytological observation of the first zygotic division confirmed the occurrence of “canonical” CI phenotypes in a fraction of the eggs. Backcross experiments demonstrated that intermediate compatibilities were not linked to host genetic background but to the *Wolbachia* strains involved. This previously unstudied intermediate penetrance CI was more severe and frequent in crosses involving *w*Pip-IV strains exhibiting *cid* variants markedly divergent from other *w*Pip groups. Our data demonstrate that CI is not always a binary compatible/incompatible phenomenon in C. pipiens but that intermediate compatibilities putatively resulting from partial mismatch due to Cid proteins divergence exist in this species complex.

## Abstract (precis)

Culex pipiens mosquitoes are infected with *w*Pip. These endosymbionts induce a conditional sterility called CI resulting from embryonic deaths, which constitutes a cornerstone for *Wolbachia* antivectorial methods.

## INTRODUCTION

In many arthropods, the fertility of two sexual partners undergoes acute reduction due to the presence of the intracellular alphaproteobacteria *Wolbachia* (1). This conditional sterility, depending on the presence of cytoplasmic factors, is called cytoplasmic incompatibility (CI). CI primarily occurs within crosses between males infected with *Wolbachia* and uninfected females, thus exhibiting reduced fertility compared to the infected ones. Such “reproductive manipulation” induced by *Wolbachia* promotes the spread of the infection (2). The loss of fertility for uninfected females, while infected females reproduce well, confers an advantage for *Wolbachia* transmission, which is the cornerstone of CI evolution. Such loss of fertility does not result from reduced egg production but from a high rate of early embryonic mortality (3, 4). Cytological embryonic observations demonstrated in *Culex*, *Drosophila*, and *Nasonia* that CI induced by *Wolbachia* is precisely due to defects in paternal chromatin during first zygotic division, suggesting a chromatin modification by some *Wolbachia* factors (5–9). Such defects during the first embryonic division can be prevented if *Wolbachia* are present in the eggs. This cytological characterization of the hallmarks of CI has contributed to the formulation of the modification-rescue (mod-resc) model that could putatively be based on toxin-antidote interactions where a toxin (the mod factor) produced by the paternal *Wolbachia* and introduced in the sperm induces embryonic mortality unless an antidote (the resc factor) is produced by the maternal *Wolbachia* in the eggs (3).

Recent studies pointed out pairs of adjacent genes called CI factors (*cif*), within the genomes of CI-inducing *Wolbachia*, as major molecular actors of CI (10–15). *Cif* is their general name, while *cid* or *cin* are specific names based on their enzymatic domains (deubiquitinase [DUB] for *cid* and nuclease for *cin* [16, 17]). The heterologous expression of either *cid* or *cin* pairs (each composed of A/B genes) in Drosophila melanogaster males induces early death for a significant number of the embryos when crossed with uninfected females (11, 12, 14). However, the abortive embryo proportion due to CI, also called CI penetrance, varies depending on the *cif* transgenes. In similar expression conditions, the *cid* genes induced stronger CI than *cin* ones (12, 14, 15). Moreover, differences in CI penetrance between the different *cid* alleles introduced in D. melanogaster have been reported: the *cidA/B^w^*^Pip^, which are secreted effectors encoded by the *w*Pip genomes from Culex pipiens (10–12), induced full CI (i.e., null hatching rate [HR], with HR equal to the proportion of hatched eggs) while *cidA/B^w^*^Mel^ factors (from the *w*Mel genome) only induced a significant decrease in HR (12, 15). Differences in CI penetrance were also reported between *w*Mel and *w*Pip, harboring different *cifA* and *cifB* genes, in the natural context of their native hosts. Indeed, in C. pipiens, all *w*Pip strains induced full CI when infected males were crossed with uninfected females (18, 19) while *w*Mel in *Drosophila* induced a partial HR reduction (12, 20). In C. pipiens, full CI occurs regardless of male age (21, 22), host genetic background (23, 24), or *Wolbachia* densities (9, 22). The cumulative presence of both functional *cid* and *cin* genes (17, 19, 25, 26) and the massive amplification-diversification of *cid* genes (9, 19, 27) provided putative genomic bases for this full CI induction. Indeed, unlike *Wolbachia* strains found in other host species where *cid* genes are monomorphic, each *w*Pip strain encodes a “repertoire” of *cid* genes, with up to 6 different variants of *cidA* and *cidB* genes in a single *Wolbachia* genome (19, 27).

The strength of *w*Pip-induced CI represents a force that certainly promoted the initial fixation and the maintenance of *w*Pip in the C. pipiens complex (28). All C. pipiens individuals are currently infected with *Wolbachia* strains belonging to the monophyletic clade of *w*Pip that is diversified into five groups, *w*Pip-I to *w*Pip-V (29). This diversity of *w*Pip strains is responsible for the unparalleled diversity of CI patterns in the C. pipiens complex described as a binary “compatible/incompatible” phenomenon (30, 31). Indeed, hundreds of crosses between C. pipiens lines from different geographical origins all infected with *w*Pip revealed the following two major outcomes based on their HR (21–23, 29, 31–34): (i) compatible crosses, with 80% ≤ mean HR ≤ 100%; in these cases, the number of unhatched eggs is similar to those of intraline crosses; or (ii) fully incompatible crosses, with null HR except for very few eggs (18, 21, 22, 34). In the latter situation, incompatibility can be either unidirectional (one cross direction is incompatible, while the reciprocal cross is compatible) or bidirectional (both cross directions are incompatible) (32, 35, 36). Reconstruction of *w*Pip phylogeny revealed that mosquitoes infected with strains from the same group are more likely to be compatible with each other, while the compatibility between host-harboring *w*Pip strains from different groups is mostly unpredictable (31). Moreover, specific variations in *cidB* repertoires harbored by males correlated with compatibility/incompatibility variations between C. pipiens lines, suggesting that some specific variants may play a strong role in this “yes-or-no” CI (19, 27). However, few cases were also reported with intermediate HR, i.e., 10% ≤ mean HR ≤ 80%, without knowing if those intermediate HR were linked to the *Wolbachia* strains involved or other factors such as nuclear incompatibilities (30, 37–43). Indeed, at the time of these intermediate HR observations, no diversity between *w*Pip strains was discovered, and it was not possible to decipher the part of nuclear genetic background versus *Wolbachia* in the observed intermediate HR.

Our recent reconstruction of *w*Pip phylogenetic groups (29, 31) and discovery of *cid* genes’ amplification and diversification led us to correlate *cid* and “yes-or-no” CI diversities in C. pipiens (19, 27). In the present study, we accurately monitored CI penetrance variations in the light of *cid* genes divergence by generating a C. pipiens compatibility matrix involving 11 lines harboring *Wolbachia* strains belonging to different *w*Pip groups (*w*Pip-I to *w*Pip-IV) and all harboring different *cid* repertoires (9, 19). This compatibility matrix is composed of estimated HR obtained from (i) 11 intraline crosses (INTRA), (ii) 12 crosses between lines harboring *w*Pip strains from the same group (INTER-INTRA), and (iii) 83 crosses between lines harboring *w*Pip from different groups (INTER-INTER). We showed, as expected, that all INTRA and INTER-INTRA (except two) crosses were fully compatible. Among the INTER-INTER crosses, 54% were totally incompatible, displaying no hatching, and 22% were considered fully compatible, while 24% of the crosses exhibited mean HRs that can be qualified as intermediate. Backcross experiments demonstrated that such intermediate HRs were not linked to host genetic background but to the *Wolbachia* strains involved. Moreover, we showed that intermediate HR values were particularly low within crosses involving *w*Pip-IV strains that also present marked phylogenetic difference in their *cid* repertoires from other *w*Pip groups (19). To visualize the developmental defects responsible for intermediate HR, we monitored the embryonic development and found defects during the first zygotic division and subsequent developmental arrest, which are typical hallmarks of “canonical CI” (9, 14). Altogether, our data demonstrate that CI is not always a “yes-or-no” phenomenon in C. pipiens but that subtle CI variations, referred to as “cryptic CI,” putatively resulting from partial mismatch due to Cif protein divergence, exist in this species complex.

## RESULTS

### HR in fully compatible crosses.

**FIG 1:**
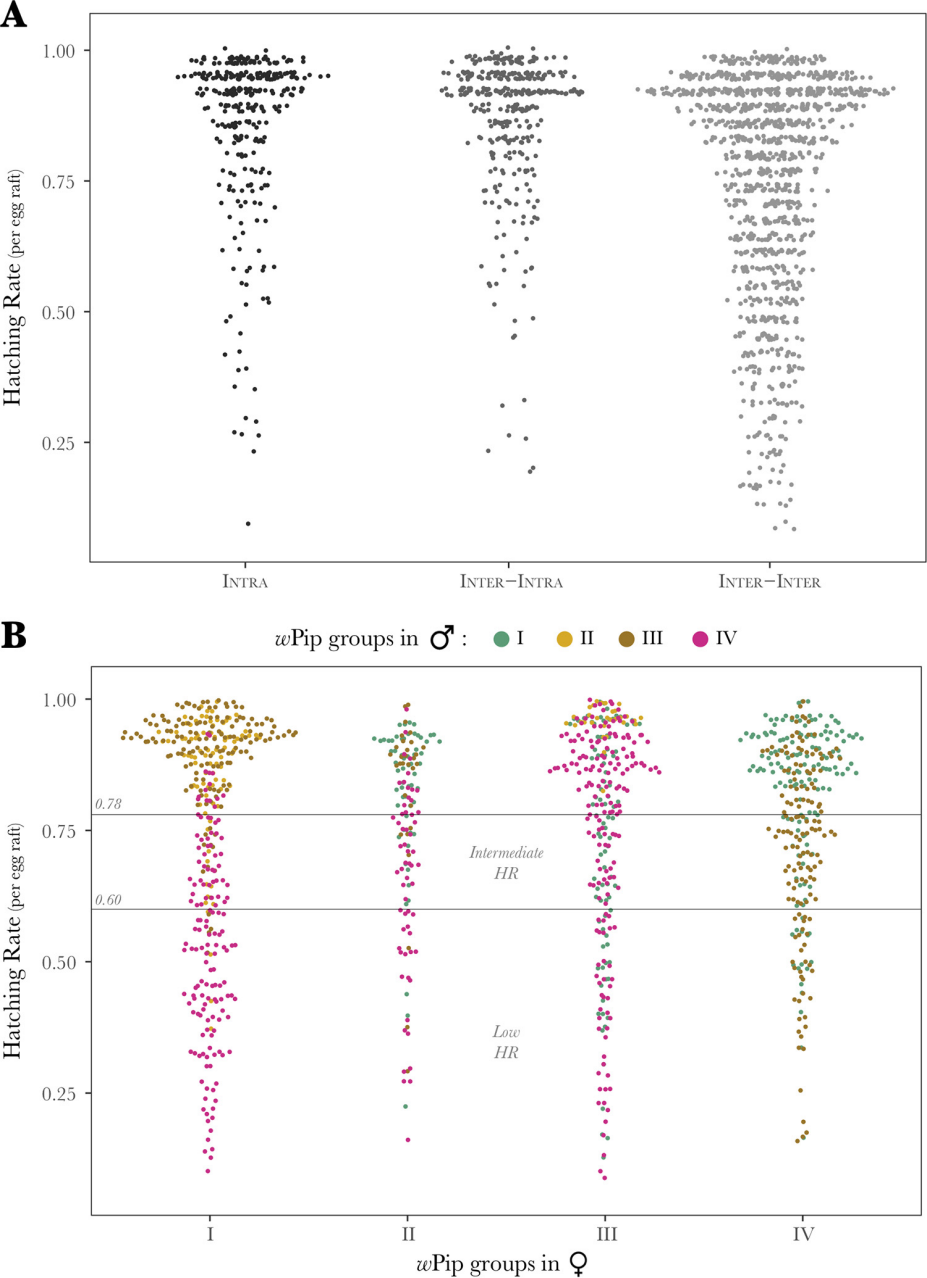
Global hatching rate comparison between INTRA, INTER-INTRA, and INTER-INTER crosses. (A) Distribution of hatching rates (HR) per egg raft in the 11 INTRA crosses (330 egg rafts; 47,504 eggs), the 12 INTER-INTRA crosses (360 egg rafts; 49,461 eggs), and the 38 INTER-INTER crosses in which eggs produced larvae (1,140 egg rafts; 169,215 eggs). (B) Influence of *w*Pip groups present in males and females on hatching rates of INTER-INTER crosses (38 different crosses; 1,140 egg rafts analyzed).

Mean HR of the 11 INTRA crosses were comprised between 0.78 and 0.95, showing that an important part of the eggs (up to 22%) failed to develop even in INTRA crosses. Intermediate HR can thus only refer to crosses with mean HR ≤78% (Fig. 1; Table S1 in the supplemental material; Data Set S1).

10.1128/mBio.02797-20.1 TABLE S1 Full matrix of crosses performed in this study. The numbers indicated are mean HR obtained for each cross on 30 eggs rafts with standard deviation. Color scale is an indication of CI penetrance in each cross (green, no CI; red, full CI). Download Table S1, XLSX file, 0.02 MB. Copyright © 2021 Sicard et al. 2021 Sicard et al. This content is distributed under the terms of the Creative Commons Attribution 4.0 International license.

10.1128/mBio.02797-20.7 DATA SET S1 Raw data for all INTRA crosses. For each egg raft, the total number of eggs, the total number of hatched larvae, and the calculated HR per egg raft are given. Download Data Set S1, XLSX file, 0.04 MB. Copyright © 2021 Sicard et al. 2021 Sicard et al. This content is distributed under the terms of the Creative Commons Attribution 4.0 International license.

### Depriving lines from *Wolbachia* did not influence INTRA HR.

To test for the effect of presence/absence of *Wolbachia*, two C. pipiens lines were tetracycline treated (SlabTC and IstanbulTC). For these “cured lines”, mean HRs were not significantly different from HRs of the corresponding INTRA crosses with infected lines (Wilcoxon *W *= 356, *P = *0.168; and *W *= 344, *P = *0.119 for Slab/SlabTC and Istanbul/IstanbulTC, respectively) (Table S1; Data Set S2).

10.1128/mBio.02797-20.8 DATA SET S2 Raw data for all crosses that involved tetracycline-treated lines. For each egg raft, the total number of eggs, the total number of hatched larvae, and the calculated HR per egg raft are given. Download Data Set S2, XLSX file, 0.02 MB. Copyright © 2021 Sicard et al. 2021 Sicard et al. This content is distributed under the terms of the Creative Commons Attribution 4.0 International license.

### No influence of host genetic backgrounds on HR.

Crosses involving females harboring the same *w*Pip strain in different genetic backgrounds (i.e. from backcrossed lines [Sl(*w*Pip-I-Tunis) and Sl(*w*Pip-IV-Harash)]) did not differ in their HRs when crossed with males from seven different lines (generalized linear models with mixed effects [GLMM]; *χ*^2^ = 2.857, degrees of freedom [df] = 1, *P = *0.091). Crosses involving males harboring the same *w*Pip strain in different genetic backgrounds showed similar HRs when crossed with females from five different lines (GLMM; *χ*^2^ = 0.414, df = 1, *P = *0.520; *χ*^2^=0.0137, df= 1, *P = *0.907; Table S1). Moreover, reciprocal crosses involving different C. pipiens species (i.e., Culex quinquefasciatus [Slab] versus C. pipiens [Istanbul]) without *Wolbachia* were not significantly different from corresponding intraspecies crosses (Wilcoxon *W *= 216, *P = *0.764 and *W* = 185, *P = *0.327, respectively; Data Set S3).

10.1128/mBio.02797-20.9 DATA SET S3 Raw data for all crosses that involved backcrossed lines. For each egg raft, the total number of eggs, the total number of hatched larvae, and the calculated HR per egg raft are given. Download Data Set S3, XLSX file, 0.04 MB. Copyright © 2021 Sicard et al. 2021 Sicard et al. This content is distributed under the terms of the Creative Commons Attribution 4.0 International license.

### INTER-INTER crosses exhibit significantly reduced HR.

The full distribution of HR per egg raft for all the crosses is presented in Fig. 1A. The mean HR (i.e., calculated on 30 egg rafts per cross) of INTRA crosses ranged from 0.78 to 0.95; the mean HR of INTER-INTRA crosses (except for two fully incompatible crosses) ranged from 0.75 to 0.93, while the mean HR of INTER-INTER crosses displayed much more variability, ranging from 0 to 0.96. Fifty-four percent (45/83) of the INTER-INTER crosses were actually fully incompatible, while 46% (38/83) produced numerous larvae (mean HR between 0.48 and 0.96). HR distributions differed significantly among the different cross types (TYPE parameter in the statistical model) that led to larval production, as follows: (i) all the 11 INTRA (330 eggs rafts analyzed for a total of 47,504 eggs), (ii) all the 12 INTER-INTRA (360 egg rafts analyzed for a total of 49,461 eggs), and (iii) 38 out of the 83 INTER-INTER (1,140 egg rafts analyzed for a total of 169,215 eggs [Fig. 1A]). HR from INTER-INTER crosses were significantly lower than others (GLMM; *χ*^2^= 8.0371, df = 2, *P = *0.018; Fig. 1A). Furthermore, the variance in HR per egg raft was significantly higher in INTER-INTER crosses (Levene’s test, *P < *0.001), while it did not differ between INTRA and INTER-INTRA (Levene’s test, *P = *0.65; Fig. 1A).

### The INTER-INTER crosses category shows a higher occurrence of intermediate HR.

Among the 38 INTER-INTER crosses in which eggs hatched (Data Set S4), 20 crosses displayed a mean HR below 78%, referred to as intermediate HR, while only 1 cross out of 12 in the INTER-INTRA showed such intermediate values. INTER-INTER crosses showed significantly more intermediate HR crosses than other types (chi-square test; *χ*^2^ = 7.346, df = 1, *P = *0.006; Table S1).

10.1128/mBio.02797-20.10 DATA SET S4 Raw data for all INTER-INTRA (between different lines that harbor *w*Pip strains for the same *w*Pip group) and INTER-INTER crosses (between lines that harbor *w*Pip strains form different *w*Pip groups). For each egg raft, the total number of eggs, the total number of hatched larvae and the calculated HR per egg raft are given. Download Data Set S4, XLSX file, 0.1 MB. Copyright © 2021 Sicard et al. 2021 Sicard et al. This content is distributed under the terms of the Creative Commons Attribution 4.0 International license.

### The lowest HRs were observed in INTER-INTER crosses involving *w*Pip-IV strains.

For the 38 INTER-INTER crosses which were not fully incompatible, global models did not reveal any significant effect of the *w*Pip group hosted by either female or male lines (GLMM; *χ*^2^ = 0.268, df = 3, *P = *0.966; *χ*^2^ = 2.742, df = 3, *P = *0.433, respectively) but pointed out a significant interaction effect between the *w*Pip groups involved in the crosses (generalized linear models [GLM]; *χ*^2^ = 113.764, df = 13, *P < *0.001; for detailed statistics, see Text S1). Careful inspection of the HR matrix revealed that 8 INTER-INTER crosses out of 38 showed a mean HR below 60%, here called low HR (Fig. 1B; Table S1). All these eight INTER-INTER crosses with low HR involved *w*Pip-IV strains (see HR per egg raft full distribution in Fig. 1B; pink dots show HR obtained in crosses involving males infected with *w*Pip-IV strains). INTER-INTER crosses with backcrossed line Sl(*w*Pip-IV-Harash) did not differ from crosses involving Harash lines (GLM; *χ*^2^ = 0.0137, df = 1, *P* = 0.907), demonstrating that it was the *w*Pip-IV strain harbored in the cytoplasm and not the host genetic background that explained such a low HR.

10.1128/mBio.02797-20.6 TEXT S1 Explanations on GLM and GLMM models performed in this study. Download Text S1, DOCX file, 0.01 MB. Copyright © 2021 Sicard et al. 2021 Sicard et al. This content is distributed under the terms of the Creative Commons Attribution 4.0 International license.

### Intermediate HR results from cryptic but canonical CI.

**FIG 2:**
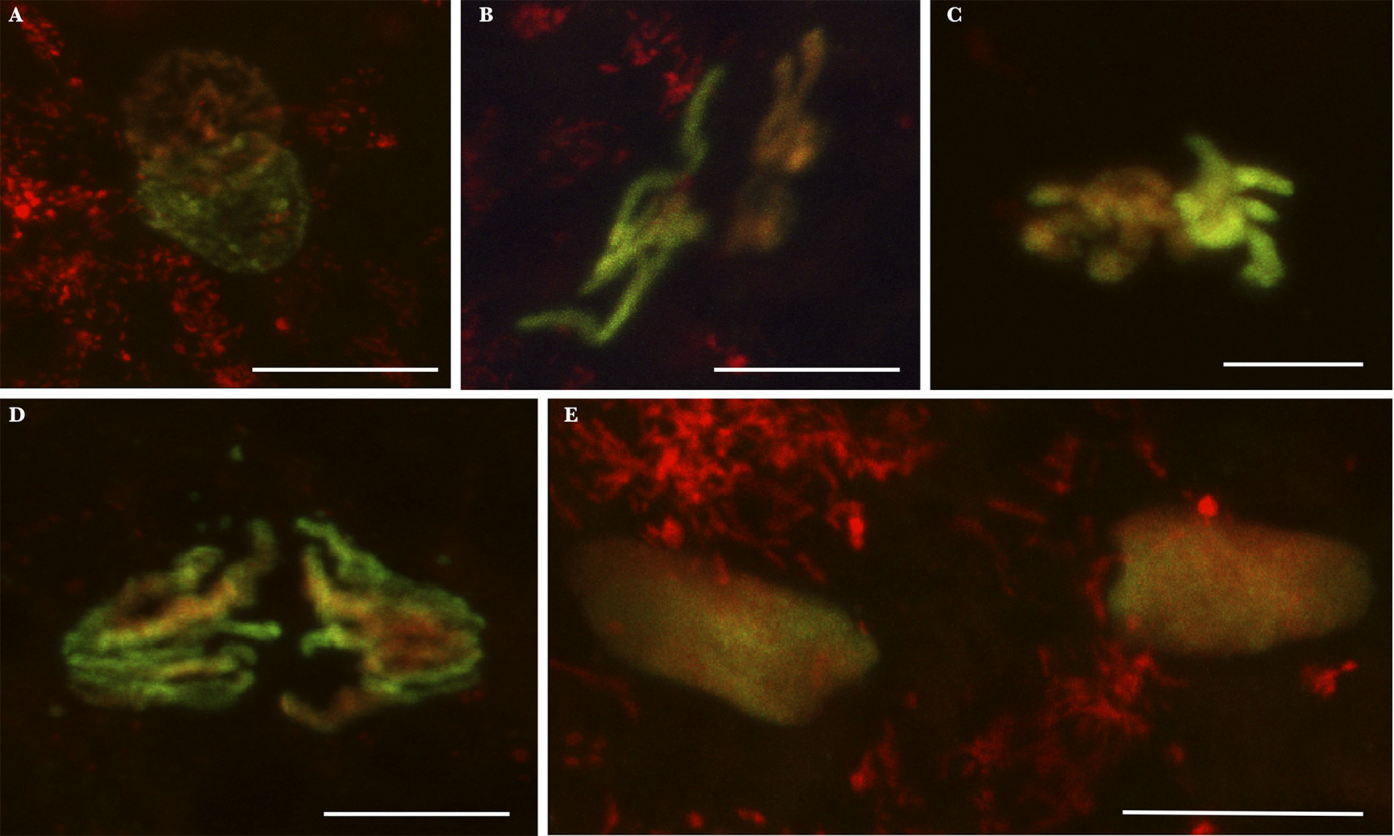
Culex pipiens embryos from INTER-INTER crosses exhibiting normal first division. Paternal chromatin appears in green/yellow (acetylated histone H4 labeling is dominant), and maternal chromatin appears in red (propidium iodide labeling is dominant). These embryos have been collected and fixed 30 min to 1 h postoviposition. (A) Apposition of maternal and paternal pronuclei; (B) chromatin under condensation; (C) condensed chromatin; (D) first mitotic division anaphase; (E) two nuclei following the first division. Scale bar represents 10 μm.

**FIG 3:**
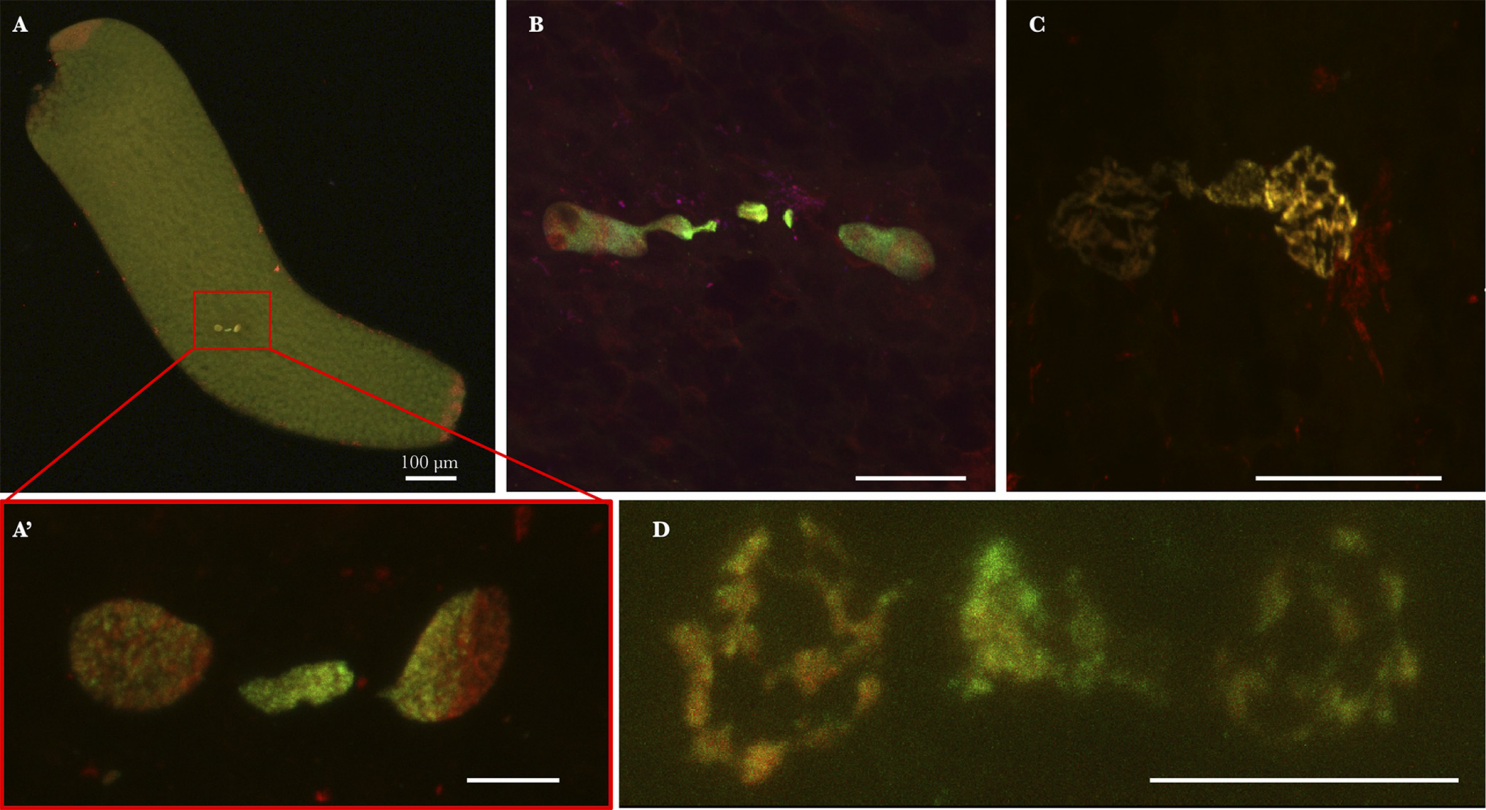
Culex pipiens embryos from INTER-INTER crosses exhibiting CI in first division. Paternal chromatin appears in green/yellow (acetylated histone H4 labeling is dominant), and maternal chromatin appears in red (propidium iodide labeling is dominant). These embryos have been collected and fixed in the first hour postoviposition. (A) Global view of a C. pipiens embryo undergoing a first mitotic division. (A′) Magnification of panel A showing paternal chromatin failed to segregate properly and form a chromatin spot between segregated nuclei. (B, C, and D) Other kinds of failed first divisions observed. Confocal stacks were obtained on embryos from several INTER-INTER crosses. Scale bar represents 10 μm.

As low HRs (mean HR under 0.6) were only observed in INTER-INTER crosses involving *w*Pip-IV strains, we (i) studied the first zygotic division resulting from these crosses, and (ii) in an attempt to quantify putative CI defects, compared them with INTER-INTRA and INTRA crosses at 5 h (Table 1 and Table S2). To verify whether intermediate HRs were due to previously described canonical CI cellular mechanisms (5–9), we visualized the first zygotic division with paternal and maternal chromatin labeled in green/yellow and red, respectively. In INTER-INTER crosses with intermediate HR, an important proportion of eggs normally hatched. Such normal embryogenesis, as documented in Fig. 2, is similar to what was observed for all INTRA embryos previously documented (9). After fertilization, maternal and paternal pronuclei migrated toward each other and apposed (documented embryos with confocal microscopy images, *n *= 3; Fig. 2A). Then, paternal and maternal chromatins condensed and entered into the first zygotic division (*n *= 1; Fig. 2B). During the first mitotic division, paternal and maternal chromosomes aligned in separate regions at the metaphase plate (*n *= 2, Fig. 2C). Both sets of chromosomes segregated equally during anaphase (*n *= 2; Fig. 2D) to produce two diploid nuclei (*n *= 1; Fig. 2E). Although our observations of first zygotic division events are not quantitative due to the technical challenge to monitor the different steps of this fast process, observations of embryos’ early development in INTER-INTER crosses with intermediate HR enabled us to document the presence of first zygotic division defects (*n *= 4; Fig. 3) that were previously observed in fully incompatible INTER-INTER crosses and absent in INTRA ones (9). As it was the green-labeled chromatin that exhibited such defects, it can be concluded that paternal chromatin is affected (Fig. 3A, A′, B, C, and D).

**TABLE 1:** Proportion of embryos that did not reach normal blastoderm stage 5 h postoviposition in one INTRA, one INTER-INTRA, and one INTER-INTER cross

Cross (male × female)	Cross type	No. of blastoderm-stage embryos	No. of embryos with abnormal development	No. of embryos with no sign of development	Total no. of embryos	% of embryos that did not reach blastoderm stage (5 h postoviposition)
Tunis × Tunis	INTRA	94	0	5	99	5
Ichkeul-13 × Harash	INTER-INTRA	46	0	1	47	2
Ichkeul-13 × SI(*w*Pip-I-Tunis)	INTER-INTER	36	5	4	45	20
Ichkeul 13 × Tunis	INTER-INTER	105	20	20	145	28

10.1128/mBio.02797-20.2 TABLE S2 Crosses performed to study cytological embryogenesis in intermediate situations. Bold letters represent crosses that were documented for developmental defects during the first zygotic division at 30 min to 1 h postoviposition; asterisk shows crosses that were compared for developmental defects at 5 h postoviposition. Download Table S2, DOCX file, 0.01 MB. Copyright © 2021 Sicard et al. 2021 Sicard et al. This content is distributed under the terms of the Creative Commons Attribution 4.0 International license.

**FIG 4:**
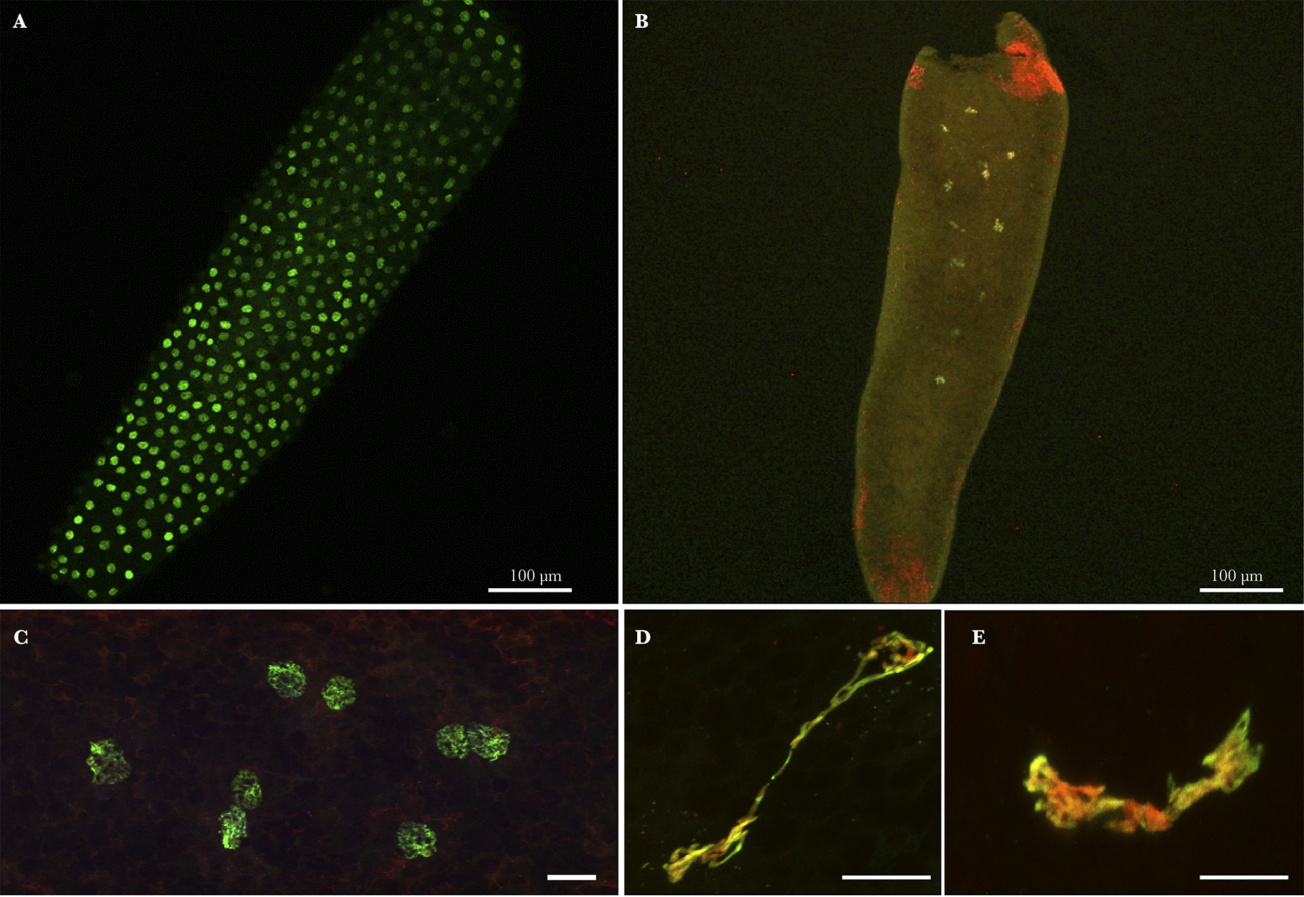
Culex pipiens embryos 5 h postoviposition in INTER-INTER crosses. Green/yellow (acetylated histone H4 labeling) and red (propidium iodide labeling). (A) Global view of a normal C. pipiens embryo having reached the expected syncytial stage. (B) Global view of an abnormal C. pipiens embryo exhibiting only few (less than 15) nuclei 5 h postoviposition. (C) Normal nuclei in a syncytial embryo. (D and E) Atypical mitotic features observed in abnormal embryos. Confocal stacks were obtained on embryos from several INTER-INTER crosses. Red dots (especially visible at the embryo’s poles in panel B) are propidium iodide-labeled *Wolbachia* in the embryo’s cytoplasm. Scale bar represents 10 μm.

For only three crosses involving *w*Pip-I and *w*Pip-IV strains (one INTRA between Tunis *w*Pip-I infected individuals; one INTER-INTRA between Ichkeul-13 *w*Pip-IV males and Harash *w*Pip-IV strain females; one INTER-INTER between Harash *w*Pip-IV males and Tunis *w*Pip-I females), we were able to produce enough observable embryos to assess the proportion of embryos with abnormal development 5 h postoviposition as presented in Table 1. At this time, embryos should have reached the syncytial blastoderm stage (∼3,200 “normal” nuclei; Fig. 4A and C), while embryos considered “abnormal” only presented few nuclei (less than 50; Fig. 4B). Moreover, atypical mitotic features were observed in these abnormal embryos (Fig. 4D and E). The proportion of abnormal embryos was less than 6% in INTRA and INTER-INTRA crosses while reaching at least 20% in the INTER-INTER cross with intermediate HR (Table 1; chi-square test; *χ*^2^ = 29.998, df = 3, *P < *0.001).

### *cid* variants from *w*Pip-IV repertoires are divergent from those of other *w*Pip groups.

**FIG 5:**
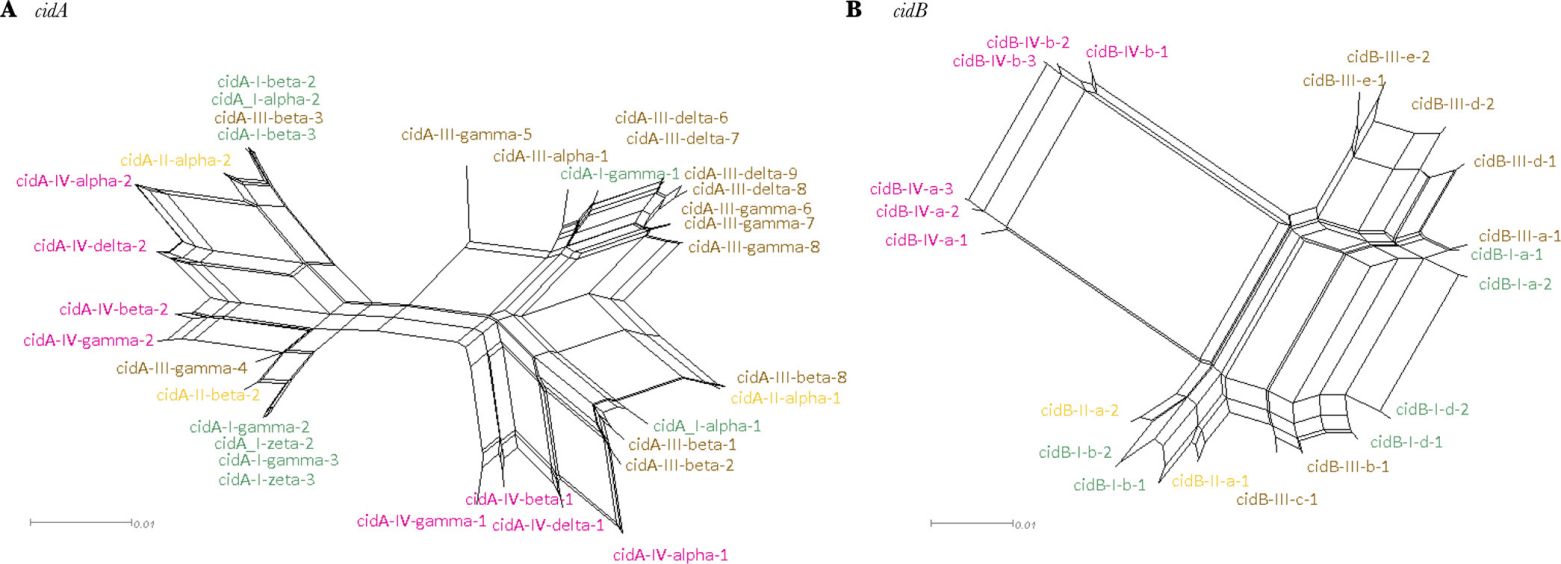
Phylogenetic networks of the *cidA* and *cidB* genes. Networks obtained with 34 *cidA* (A) and 21 *cidB* (B) nucleotide variants present in the repertoires of the 11 strains from the four phylogenetic *w*Pip groups studied here. The networks were obtained using the neighbor-net method. Each edge (or set of parallel edges) corresponds to a split in the data set and has a length equal to the weight of the split. Incompatible splits produced by recombination are represented by boxes in the network. *w*Pip-I *cid* variants are in green, *w*Pip-II *cid* variants are in yellow, *w*Pip-III *cid* variants in brown, and *w*Pip-IV *cid* variants are in pink.

The phylogenetic *cidA* and *cidB* networks constructed with *w*Pip strains repertoires showed that *w*Pip strains from the *w*Pip-IV group exhibited markedly divergent *cidA* and *cidB* variants. For both *cidA* and *cidB* variants, *w*Pip-IV variants clustered remotely from other groups’ variants (Fig. 5; Tables S3 and S4). Two well-separated clusters of *w*Pip-IV *cidA* variants appeared on the network, while all *cidB* variants clustered altogether (Fig. 5). For other *w*Pip groups, no clear *w*Pip-group-based clustering was observed (Fig. 5).

10.1128/mBio.02797-20.3 TABLE S3 *cidA* and *cidB* repertoires of the *w*Pip strains studied here. Each *w*Pip strain harbors a specific combination of different *cidA* and *cidB* variants in its genome. Each combination is called a repertoire. Download Table S3, DOCX file, 0.02 MB. Copyright © 2021 Sicard et al. 2021 Sicard et al. This content is distributed under the terms of the Creative Commons Attribution 4.0 International license.

10.1128/mBio.02797-20.4 TABLE S4 Accession numbers for all *cidA* and *cidB w*Pip variants sequenced to date. All these variants were included in phylogenetic networks analyses. Download Table S4, DOCX file, 0.02 MB. Copyright © 2021 Sicard et al. 2021 Sicard et al. This content is distributed under the terms of the Creative Commons Attribution 4.0 International license.

## DISCUSSION

In arthropods in which CI is mainly studied between infected males and uninfected females, including major insect models such as *Drosophila* and *Nasonia*, CI penetrance was proved to depend on *Wolbachia* strains, their densities, host genetic background, age of the males, and environmental factors such as temperature (44–59). On the contrary, in Culex pipiens s.l. mosquitoes, these factors did not affect CI penetrance (19, 22–24, 33): full CI (hatching rate [HR], 0) is reported between infected males and uninfected females (cured of *Wolbachia* with antibiotics) whatever their geographical origin, age, or genetic background (9, 18, 23, 33). However, hundreds of crosses between lines infected with *Wolbachia* revealed unparalleled variations in CI patterns in C. pipiens. Two main opposite outcomes were observed: either the crosses were compatible (mean HR ≥ 80%) or incompatible, producing almost no larvae (mean HR < 0.01%) (18, 21–23, 31, 32, 34, 40). Early in the study of CI in *Culex*, backcross experiments demonstrated that the host genome does not influence the outcome of a given cross (9, 24, 32, 60). In the present study, we conducted backcross experiments for two of our lines and also performed crosses between cured individuals from different C. pipiens species, which again confirmed that host genetic background does not impact compatibility.

Most crosses and backcrosses showed that CI in C. pipiens is a binary compatible/incompatible phenotype under the sole control of *Wolbachia*. However, in the numerous articles that presented results of interline C. pipiens crosses from different parts of the world, rare cases of intermediate HR were reported (30, 37–43). At the time of these publications, all the *w*Pip were considered clonal due to monomorphic genetic markers available (34, 61). Intermediate HRs were thus attributed to putative undiscovered *Wolbachia* variability (including different *w*Pip sublines in the same laboratory line) and most probably to putative host “restorer” nuclear factors counteracting *Wolbachia* CI induction (40, 41, 62). In the present paper, we investigated these intermediate HR situations in light of our present knowledge of *w*Pip genomes (19, 27, 29, 31). To that extent, we studied 106 crosses between 11 C. pipiens isofemale lines infected with different *w*Pip strains from different groups (I to IV), each exhibiting different *cidA-cidB* repertoires (9, 19) (Table S3 in the supplemental material). Different types of crosses were performed, including (i) INTRA crosses between mosquitoes from the same line, (ii) INTER-INTRA crosses between mosquitoes infected with different strains from the same *w*Pip group, and (iii) INTER-INTER crosses between mosquitoes infected with different *w*Pip groups.

For the 11 INTRA crosses performed in this study, mean HRs were all comprised between 78% and 95%, showing that a significant proportion of eggs never hatched even in fully compatible crosses. Previous cytological observations of C. pipiens early development in seven INTRA crosses with or without *Wolbachia* (i.e., after antibiotics treatment) did not detect any CI typical defects (9). Here, we reported no difference in HR in the same lines with or without *Wolbachia*, confirming that CI induced by *Wolbachia* is not responsible for the 5% to 22% of the eggs that did not reach the larval stage. Abortive eggs in INTRA crosses certainly resulted from imperfect fertilization and/or intrinsic mortality during development from eggs to larvae (9, 18, 40). The 12 INTER-INTRA crosses, involving lines from different locations but harboring the same *w*Pip group, exhibited HRs similar to INTRA crosses, except for two cases of unidirectional incompatibility, again demonstrating that the *w*Pip group is a major predictor of compatibility between C. pipiens lines (Fig. 1A; Table S1) (31).

Heterogeneity in compatibility clearly increased in INTER-INTER crosses (Table S1). Among the 83 performed here, we found that 54% of them were fully incompatible, while the other 46% (38/83) were fertile and exhibited HR comprised between 48% and 96%. Global HR statistical analyses, including all fertile crosses (11 INTRA, 12 INTER-INTRA, and 38 INTER-INTER crosses) showed that HR was significantly lower in INTER-INTER crosses and that variance in HRs among egg rafts was significantly higher in INTER-INTER than INTER-INTRA and INTRA crosses (Fig. 1A). Moreover, we found that 53% of the fertile INTER-INTER crosses actually exhibited HRs that were low enough to be characterized as intermediate. We also found that the interaction between the *w*Pip groups infecting the male and female lines significantly influenced HR. Careful inspection of the HR matrix revealed that the crosses with a low HR below 60% (8 crosses out of the 20 with intermediate HR) were only observed in INTER-INTER crosses involving *w*Pip-IV strains (Fig. 1B and Table S1). Atyame et al. (31) had already shown that *w*Pip-IV group-infected C. pipiens lines exhibited markedly different crossing types from lines infected with other *w*Pip groups. Network phylogenetic analyses of all the 34 *cidA* and 21 *cidB* different variants characterized in the *w*Pip strains studied here revealed that *cid*-IV variants (especially *cidB*) were divergent, gathering in specific clusters, while other *w*Pip groups are mixed altogether. This suggests that Cid proteins that are considered major effectors of CI (15, 17) are divergent in *w*Pip-IV strains compared to other *w*Pip groups (Fig. 5).

To investigate whether intermediate HR resulted from canonical CI, i.e., paternal chromatin defects during first zygotic division, we monitored the first stages of embryonic development in embryos from INTER-INTER crosses. In these crosses, even with low HR, many embryos exhibited normal development into larvae (Fig. 2). However, in a few embryos, we were able to document imperfect paternal chromatin segregation during the first zygotic division (Fig. 3). These embryonic defects, which were never observed in INTRA crosses (9), were similar to those reported in fully incompatible crosses (9). Such defects in the first zygotic division likely produced aneuploid nuclei which might disrupt further development or even arrest the embryogenesis. The proportion of embryos that did not reach blastoderm stage 5 h postoviposition, but presented instead few nuclei only, can be considered a quantitative proxy for the occurrence of CI defects during the first division. We observed a larger amount of abnormal developmental stages, 5 h postoviposition, in INTER-INTER crosses than the INTRA and INTER-INTRA crosses (Table 1). Abnormal embryos, which represented 20% of the embryos in the INTER-INTER crosses studied and 5% in the INTRA one (Table 1), displayed very few (or no) nuclei (Fig. 4B). These observations suggest that embryonic defects during the first division are responsible for the intermediate HR observed in the analyzed INTER-INTER cross (Table 1). The intermediate HR observed in INTER-INTER crosses could be attributed to cryptic CI (in that it has a weak penetrance) but canonical CI (in that it translates into the same cytological defects).

In the light of the toxin-antidote model of CI, penetrance would depend on the interaction between CidA, CidB, and their specific substrates, eventually leading to paternal chromatin defects or its rescue (15, 16, 63). In C. pipiens, as all *w*Pip genomes encode a repertoire of several polymorphic variants of CidA and CidB (19, 27), full compatibility could result from multiple interactions between different CidA and CidB variants even in INTRA or INTER-INTRA crosses. In every C. pipiens male, several CidB proteins differing in their amino acid sequences might be introduced in the sperm and then in the egg during fertilization where several CidA proteins might also be present. Full compatibilities reported here in some INTER-INTER crosses involving different *w*Pip groups with totally different CidA/CidB repertoires (Fig. 1) suggest (i) that strict specific interactions between cognate variants are not required for full compatibility, and (ii) a potential redundancy in the interaction between CidA/CidB variants. The intermediate HR resulting from cryptic CI in a given INTER-INTER cross can hypothetically result from partial rescue due to imperfect interactions between the CidA and the CidB from the two *w*Pip strains repertoires. Since most of the embryos from intermediate HR crosses developed into living larvae, it certainly means that, in those individuals, CidB toxicity has been efficiently counteracted. On the contrary, in embryos exhibiting CI, CidB toxicity would not have been counteracted properly. This heterogeneity could be explained if embryo rescue depends on one or a few matching CidA variants which might be required in a larger quantity for the rescue to occur. However, it is possible that in certain eggs, the expression of the(se) CidA variant(s) would be too low to counteract the CidB toxicity. This would be especially true for neutralizing CidB proteins encoded by *w*Pip-IV strains that show striking differences in their sequences from other *w*Pip groups (Fig. 5). Less efficacy in the interactions between CidB-IV proteins and CidA from other groups could explain their higher probability to be involved in both (i) full incompatibility as reported in reference 31, and (ii) cryptic CI as reported here.

The interactions between the CidA and CidB repertoires encoded by *w*Pip strains determine the developmental fate of each embryo of a given cross, normal development versus CI. CI penetrance (i.e., the proportion of embryos undergoing CI) in a given cross could then be determined by the diversity of *cidA/cidB* genes of the different *w*Pip genomes hosted by the different C. pipiens lines, their expression levels, and the affinity between the resulting proteins.

## MATERIALS AND METHODS

### Culex pipiens lines.

Eleven isofemale lines were used (Table S5 in the supplemental material). They differed in (i) their geographical origins, (ii) the species they belong to, (iii) the *w*Pip group (I, II, III, or IV), and (iv) their *cid* repertoires. The *Wolbachia* group was checked by performing a pk1 PCR-restriction fragment length polymorphism (RFLP) test (64) on DNA extracted using cetyltrimethylammonium bromide (CTAB) protocol (65). Tetracycline-treated *Wolbachia*-free lines (TC lines), named SlabTC and IstanbulTC, were obtained from Slab- and Istanbul-infected lines as described in reference 33. The absence of *Wolbachia* was checked by PCR on a fragment of the *wsp* gene using the primers designed in reference 66. TC-treated lines were raised at least four generations without tetracycline before experiments. The *w*Pip-I strain from the Tunis line and *w*Pip-IV strain from the Harash line were independently introgressed into the Slab line nuclear genetic background through 8 backcrosses as described in reference 9.

10.1128/mBio.02797-20.5 TABLE S5 Information on the *Culex* lines and the *Wolbachia* strains studied. Download Table S5, DOCX file, 0.02 MB. Copyright © 2021 Sicard et al. 2021 Sicard et al. This content is distributed under the terms of the Creative Commons Attribution 4.0 International license.

### Hatching rate estimations.

To test for a putative effect of *Wolbachia* on basic intraline HR, we performed two intraline crosses between males and females from cured lines (SlabTC and IstanbulTC) and compared them with infected intraline crosses (Slab and Istanbul, respectively). To test for a putative impact of the different *w*Pip groups on HR, we carried out 106 different crosses between males and females, including (i) from the same line (11 intraline crosses, called INTRA crosses), (ii) from two distinct mosquito lines infected with *w*Pip strains from the same group (12 interline-intragroup crosses, called INTER-INTRA crosses), and (iii) from two distinct mosquito lines infected with *w*Pip strains from different groups (83 interline-intergroup crosses, called INTER-INTER crosses).

To test for a potential impact of host genetic background on HR, we performed 23 extra crosses involving the two backcrossed lines [Sl(*w*Pip-I-Tunis) or Sl(*w*Pip-IV-Harash)]. Moreover, to study the putative effect of interspecies crosses (i.e., C. pipiens versus C. quinquefasciatus) on HR, we performed the reciprocal crosses between SlabTC (C. quinquefasciatus) and IstanbulTC (C. pipiens) lines. To perform each of these 135 different crosses, 2-day-old males (*n* = 50) and females (*n* = 100) were put together in cages. After 6 days, females were blood fed with turkey blood using a Hemotek feeding system (Discovery Workshops). After 5 days, egg rafts were collected. After the death of all the larvae (i.e., about 5 days after hatching), pictures of both eggs and larvae for 30 egg rafts per cross were taken. Eggs and larvae were counted manually on ImageJ (67). HR was calculated per egg raft as the ratio between the total number of larvae and the total number of eggs.

### Cellular study of embryogenesis.

To search for putative embryonic defects that might confirm the involvement of canonical CI in INTER-INTER crosses resulting in intermediate HR, several crosses involving males from different lines infected with *w*Pip-IV strains were performed (Table S2). To that extent, cages containing 50 males and 100 females were put into a closet where the day-night cycle was inverted to allow collection of eggs during the day. After 6 days in these cages, females were fed with turkey blood, and waterpots were placed into the cages for 30 min to 1 h to allow females to lay egg rafts. For C. pipiens embryos, at 25°C, the meiosis is approximately completed 30 min postoviposition and the first mitotic nucleus division 15 min after, while 5 h after oviposition, the embryos reach the blastoderm stage (9). Freshly collected eggs (30 min to 5 h) were fixed, dechorionated, and observed as previously described in reference 9.

### Statistical analysis.

We used generalized linear models (GLM) or generalized linear models with mixed effects (GLMM) with a logit link function (see Text S1). To test for potential impact of *Wolbachia* presence/absence and host species, Wilcoxon tests were performed (68). To compare the proportion of (i) intermediate HR between different types (INTRA, INTER-INTRA, and INTER-INTER), and (ii) abnormal embryos between INTRA, INTER-INTRA, and INTER-INTER crosses, *χ*^2^ tests were performed. The differences in variance among INTRA, INTER-INTRA, and INTER-INTER crosses were analyzed using Levene’s test (69). All computations were performed using R version 3.4.4 (70).

### Phylogenetic networks of the *cidA* and *cidB* genes.

All the *cidA* and *cidB* repertoires of the *Wolbachia* strains hosted by the 11 crossed lines were already published except for Brazil that has been obtained by PCR cloning followed by Sanger sequencing as previously described in references 9 and 19. Sequenced variants (accession numbers given in Table S4) were aligned using the Muscle algorithm implemented in SeaView 6.4.1 software (71) and then analyzed within a phylogenetic network framework from uncorrected P distances by the neighbor-net method implemented in SplitsTree4 (72) to account for potentially conflicting signals due to recombination.
