# Supplementary material for: Cytoplasmic Incompatibility Variations in Relation with Wolbachia cid Genes Divergence in Culex pipiens
Source: mBio. 2021 Feb 9;12(1):e02797-20. doi: 10.1128/mBio.02797-20 (PMC7885119; doi:10.1128/mBio.02797-20)
Supplement: TEXT S1 [file mBio.02797-20-s0001.docx]

**Text S1: Explanations on GLM and GLMM models performed in this study**

Variability of Hr among crosses was analyzed using a GLM: Hr = Cross + ε, with Hr for each cross with ‘Cross’ the interaction between males (Mal) and females (Fem) from different lines and ε the error parameter, following a binomial distribution.

To test for the specific effect of Mal, Fem and Type (i.e. the Intra, Inter-Intra and Inter-Inter type of cross) separately, GLMMs were used: Hr = Male + Female + Type + 1|Cross + ε with Male and Female respectively the Mal and Fem lines involved in each cross and Type as fixed effects, with Cross (the interaction between Mal and Fem lines) as a random effect (as crosses to produce embryos necessarily require an interaction between females and males), and ε the error parameter, following a binomial distribution.

To test if the interaction between the different *w*Pip groups in the Mal and Fem lines could affect the hatching rate, we performed the GLM: Hr = Comb + ε with Comb the interaction between the different *w*Pip group infecting the Mal line and the Fem line respectively and ε the error parameter, following a binomial distribution. To test for specific effect of *w*Pip group in Mal or Fem lines the following GLMM was performed: Hr = *W*gm + *W*gf + 1|Comb + ε with *W*gm (the group of *w*Pip in the Mal line), *W*gf (the group of *w*Pip in the Fem line) and Comb as a random effect and ε the error parameter, following a binomial distribution.

We tested for a specific effect of the paternal host genetic background (MalBack) in crosses involving males from Sl(*w*PipI-Tunis) and Tunis lines or males from Sl(*w*PipIV-Harash) and Harash lines which host respectively the same *Wolbachia* strain in two different genetic background, using a GLMM: Hr = MalBack + Female + 1|Cross + ε with Cross as a random effect, and ε the error parameter, following a binomial distribution.

To test for a specific effect of the maternal host genetic background in crosses involving females from Sl(wPipIV-Harash) and Harash lines which host the same *Wolbachia* in two different genetic background we used two GLMM : Hr = FemBack + male + 1|Cross + ε with Cross as a random effect, and with ε the error parameter, following a binomial distribution.
