# Supplementary material for: Cytoplasmic Incompatibility Variations in Relation with Wolbachia cid Genes Divergence in Culex pipiens
Source: mBio. 2021 Feb 9;12(1):e02797-20. doi: 10.1128/mBio.02797-20 (PMC7885119; doi:10.1128/mBio.02797-20)
Supplement: TABLE S2 [file mBio.02797-20-st002.docx]

| **Intra cross** | **Inter-Intra cross** | **Inter-Inter crosses** |
| --- | --- | --- |
| ♂ Tunis x ♀ Tunis* | ♂ Ichkeul 13 x ♀ Harash* | **♂ Harash x ♀ Tunis** (Fig2 A, B, C, D, E; Fig3 B, C; Fig4 E) |
|  |  | **♂ Harash x ♀ Slab(*w*PipI-Tunis)** (Fig 3D) |
|  |  | **♂ Harash x ♀ Utique** (Fig4 D) |
|  |  | ♂ Ichkeul 13 x ♀ Tunis* |
|  |  | ♂ Ichkeul 13 x ♀ Slab(*w*PipI-Tunis)* |

Table S2: Crosses performed for cytological embryogenesis in ‘intermediate’ situations

In bold letters crosses that were documented for developmental defects during the first zygotic division at 30 min to 1H post-oviposition

* Crosses that were compared for developmental defects at 5H post-oviposition
