# Supplementary material for: Cytoplasmic Incompatibility Variations in Relation with Wolbachia cid Genes Divergence in Culex pipiens
Source: mBio. 2021 Feb 9;12(1):e02797-20. doi: 10.1128/mBio.02797-20 (PMC7885119; doi:10.1128/mBio.02797-20)
Supplement: TABLE S3 [file mBio.02797-20-st003.docx]

Table S3: *cidA* and *cidB* repertoires of the *w*Pip strains studied here. Each wPip strain harbors a specific combination of different *cidA* and *cidB* variants in their genomes. This combination is called a repertoire.

| *w*Pip strains | Group | *cidA* | *cidB* | Reference |
| --- | --- | --- | --- | --- |
| Tunis | I | I-α1, I-β2, I-γ1, I-γ2 | I-a1, I-a2, I-b1, I-b2 | (1) |
| Utique | I | I-α1, I-α2, I-γ1, I-γ2, I-ζ3 | I-b1, I-b2 | (2) |
| Brazil | I | I-α1, I-β2, I-β3, I-γ1, I-γ3, I-ζ2 | I-a1, I-a2, I-b1, I-b2, I-d1, I-d2 | This study |
| Lavar | II | II-α1, II-α2, II-β2 | II-a1, II-a2 | (1) |
| Slab | III | III-β8, III-γ4, III-γ5, III-γ6, III-γ7, III-γ8, III-δ6, III-δ7, III-δ8, III-δ9 | III-d1, III-d2, III-e1, III-e2 | (2) |
| Maclo | III | III-α1, III-β1, III-β2, III-β3 | III-a1, III-b1, III-c1 | (1) |
| Istanbul | IV | IV-α1, IV-α2, IV-γ1, IV-γ2, IV-δ1, IV-δ2 | IV-a1, IV-a2, IV-b1, IV-b2 | (1) |
| Harash | IV | IV-α1, IV-α2, IV-β1, IV-β2 | IV-a1, IV-a3, IV-b1, IV-b3 | (1) |
| Ich09 | IV | IV-α1, IV-α2, IV-δ1, IV-δ2 | IV-a1, IV-a2 | (1) |
| Ich13 | IV | IV-α1, IV-α2, IV-β1, IV-β2, | IV-a3, IV-b3 | (1) |
| Ich21 | IV | IV-α1, IV-α2, IV-δ1, IV-δ2 | IV-a1, IV-a2, IV-a3, IV-b1, IV-b3 | (1) |

1. Bonneau M, Atyame CM, Beji M, Justy F, Cohen-Gonsaud M, Sicard M, Weill M. 2018. *Culex pipiens* crossing type diversity is governed by an amplified and polymorphic operon of *Wolbachia*. Nat Commun 9:1–10.

2. Bonneau M, Landmann F, Labbé P, Justy F, Weill M, Sicard M. 2018. The cellular phenotype of cytoplasmic incompatibility in *Culex pipiens* in the light of cidB diversity. PLoS Pathog 14:1–25.
