# Supplementary material for: Cytoplasmic Incompatibility Variations in Relation with Wolbachia cid Genes Divergence in Culex pipiens
Source: mBio. 2021 Feb 9;12(1):e02797-20. doi: 10.1128/mBio.02797-20 (PMC7885119; doi:10.1128/mBio.02797-20)
Supplement: TABLE S4 [file mBio.02797-20-st004.docx]

| *cidA* | **Variant sequence submission name** | **Accession number** | *cidB* | **Variant sequence submission name** | **Accession number** |
| --- | --- | --- | --- | --- | --- |
|  | cidA_I(alpha/1) | MF444963 |  | cidB_I(a/1) | MF444982 |
|  | cidA_I(gamma/1) | MF444964 |  | cidB_I(a/2) | MF444983 |
|  | cidA_I(gamma/2) | MF444965 |  | cidB_I(b/1) | MF444984 |
|  | cidA_I(beta/2) | MF444966 |  | cidB_I(d/1) | MW315163 |
|  | cidA_I(beta/3) | MW315160 |  | cidB_I(d/2) | MW315164 |
|  | cidA_I(gamma/3) | MW315161 |  | cidB_I(b/2) | MF444985 |
|  | cidA_I(zeta/3) | MW315162 |  | cidB_II(a/2) | MF444986 |
|  | cidA_I(alpha/2) | MH544806 |  | cidB_II(a/1) | MF444987 |
|  | cidA_I(zeta/2) | MH544808 |  | cidB_III(c/1) | MF444988 |
|  | cidA_II(alpha/1) | MF444967 |  | cidB_III(a/1) | MF444989 |
|  | cidA_II(alpha/2) | MF444968 |  | cidB_III(b/1) | MF444990 |
|  | cidA_II(beta/2) | MF444969 |  | cidB_III(d/1) | MH544820 |
|  | cidA_III(alpha/1) | MF444970 |  | cidB_III(d/2) | MH544821 |
|  | cidA_III(beta/2) | MF444971 |  | cidB_III(e/1) | MH544822 |
|  | cidA_III(beta/1) | MF444972 |  | cidB_III(e/2) | MH544823 |
|  | cidA_III(beta/3) | MF444973 |  | cidB_IV(a/1) | MF444991 |
|  | cidA_III(beta/8) | MH544810 |  | cidB_IV(a/2) | MF444992 |
|  | cidA_III(gamma/4) | MH544811 |  | cidB_IV(b/1) | MF444993 |
|  | cidA_III(gamma/5) | MH544812 |  | cidB_IV(b/2) | MF444994 |
|  | cidA_III(gamma/6) | MH544813 |  | cidB_IV(a/3) | MF444995 |
|  | cidA_III(gamma/7) | MH544814 |  | cidB_IV(b/3) | MF444996 |
|  | cidA_III(gamma/8) | MH544815 |  |  |  |
|  | cidA_III(delta/6) | MH544816 |  |  |  |
|  | cidA_III(delta/7) | MH544817 |  |  |  |
|  | cidA_III(delta/8) | MH544818 |  |  |  |
|  | cidA_III(delta/9) | MH544819 |  |  |  |
|  | cidA_IV(alpha/1) | MF444974 |  |  |  |
|  | cidA_IV(alpha/2) | MF444975 |  |  |  |
|  | cidA_IV(gamma/1) | MF444976 |  |  |  |
|  | cidA_IV(gamma/2) | MF444977 |  |  |  |
|  | cidA_IV(delta/1) | MF444978 |  |  |  |
|  | cidA_IV(delta/2) | MF444979 |  |  |  |
|  | cidA_IV(beta/1) | MF444980 |  |  |  |
|  | cidA_IV(beta/2) | MF444981 |  |  |  |
