# Supplementary material for: Cytoplasmic Incompatibility Variations in Relation with Wolbachia cid Genes Divergence in Culex pipiens
Source: mBio. 2021 Feb 9;12(1):e02797-20. doi: 10.1128/mBio.02797-20 (PMC7885119; doi:10.1128/mBio.02797-20)
Supplement: TABLE S5 [file mBio.02797-20-st005.docx]

| Line | *Wolbachia* group | *Culex* species | Country | Year of collection | Reference |
| --- | --- | --- | --- | --- | --- |
| Tunis | *w*PipI | *pipiens* | Tunisia | 1995 | (1) |
| Utique | *w*PipI | *pipiens* | Tunisia | 2014 | (2) |
| Brazil | *w*PipI | *quinquefasciatus* | Brazil | 2000 | This study* |
| Lavar | *w*PipII | *pipiens* | France | 2003 | (3) |
| Slab | *w*PipIII | *quinquefasciatus* | USA | 1954 | (4) |
| Maclo | *w*PipIII | *quinquefasciatus* | USA | 1984 | (5) |
| Istanbul | *w*PipIV | *pipiens* | Turkey | 2003 | (3) |
| Harash | *w*PipIV | *pipiens* | Algeria | 2006 | (6) |
| Ichkeul 09 | *w*PipIV | *pipiens* | Tunisia | 2010-2011 | (7) |
| Ichkeul 13 | *w*PipIV | *pipiens* | Tunisia | 2010-2011 | (7) |
| Ichkeul 21 | *w*PipIV | *pipiens* | Tunisia | 2010-2011 | (7) |

Table S5: Information on the *Culex pipiens* lines and the *Wolbachia* strains studied

*The original capture was at Sao Paulo City. Brazil came at ISEM in 2015 from lab colony that was in Pr M Capurro lab at the University of Sao Paulo for 15 years. An isofemale line was then established from samples received from the colony.

1. Ben Cheikh H, Ben Ali-Haouas Z, Marquine M, Pasteur N. 1998. Resistance to Organophosphorus and Pyrethroid Insecticides in *Culex pipiens* (Diptera: Culicidae) from Tunisia. J Med Entomol 35:251–260.

2. Bonneau M, Landmann F, Labbé P, Justy F, Weill M, Sicard M. 2018. The cellular phenotype of cytoplasmic incompatibility in *Culex pipiens* in the light of cidB diversity. PLoS Pathog 14:1–25.

3. Duron O, Lagnel J, Raymond M, Bourtzis K, Fort P, Weill M. 2005. Transposable element polymorphism of *Wolbachia* in the mosquito *Culex pipiens*: evidence of genetic diversity, superinfection and recombination. Mol Ecol 14:1561–1573.

4. Georghiou GP, Metcalf RL, Gidden FE. 1966. Carbamate-resistance in mosquitos. Bull World Health Organ 35:691–708.

5. Duron O, Fort P, Weill M. 2006. Hypervariable prophage WO sequences describe an unexpected high number of *Wolbachia* variants in the mosquito *Culex pipiens*. Proc R Soc B 273:495–502.

6. Alout H, Labbé P, Berthomieu A, Pasteur N, Weill M. 2009. Multiple duplications of the rare *ace-1* mutation F290V in *Culex pipiens* natural populations. Insect Biochem Mol Biol 39:884–891.

7. Bonneau M, Atyame CM, Beji M, Justy F, Cohen-Gonsaud M, Sicard M, Weill M. 2018. *Culex pipiens* crossing type diversity is governed by an amplified and polymorphic operon of *Wolbachia*. Nat Commun 9:1–10.
